# The iTreAD project: a study protocol for a randomised controlled clinical trial of online treatment and social networking for binge drinking and depression in young people

**DOI:** 10.1186/s12889-015-2365-2

**Published:** 2015-10-06

**Authors:** F. J. Kay-Lambkin, A. L. Baker, J. Geddes, S. A. Hunt, K. L. Woodcock, M. Teesson, C. Oldmeadow, T. J. Lewin, B. M. Bewick, K. Brady, B. Spring, M. Deady, E. Barrett, L. Thornton

**Affiliations:** NHMRC Centre for Research Excellence in Mental Health and Substance Use, National Drug and Alcohol Research Centre, University of New South Wales, Sydney, Australia; Priority Research Centre for Translational Neuroscience and Mental Health, The University of Newcastle, Callaghan, Australia; Clinical Research Design, Information, Technology and Statistical Support, Hunter Medical Research Institute, New Lambton Heights, Australia; School of Medicine, Leeds Institute of Health Sciences, Faculty of Medicine and Health, University of Leeds, Leeds, UK; Psychiatry and Behavioral Sciences, Medical University of South Carolina, Charleston, USA; Center for Behavior and Health, Institute for Public Health and Medicine, Northwestern University, Evanston, USA

**Keywords:** Social networking, Internet treatment, Depression, Binge drinking, Young people

## Abstract

**Background:**

Depression and binge drinking behaviours are common clinical problems, which cause substantial functional, economic and health impacts. These conditions peak in young adulthood, and commonly co-occur. Comorbid depression and binge drinking are undertreated in young people, who are reluctant to seek help via traditional pathways to care. The iTreAD project (internet Treatment for Alcohol and Depression) aims to provide and evaluate internet-delivered monitoring and treatment programs for young people with depression and binge drinking concerns.

**Methods:**

Three hundred sixty nine participants will be recruited to the trial, and will be aged 18–30 years will be eligible for the study if they report current symptoms of depression (score 5 or more on the depression subscale of the Depression Anxiety Stress Scale) and concurrent binge drinking practices (5 or more standard drinks at least twice in the prior month). Following screening and online baseline assessment, participants are randomised to: (a) online monthly self-assessments, (b) online monthly self-assessments + 12-months of access to a 4 week online automated cognitive behaviour therapy program for binge drinking and depression (DEAL); or (c) online monthly assessment + DEAL + 12-months of access to a social networking site (Breathing Space). Independent, blind follow-up assessments occur at 26, 39, 52 and 64-weeks post-baseline.

**Discussion:**

The iTreAD project is the first randomised controlled trial combining online cognitive behaviour therapy, social networking and online monitoring for young people reporting concerns with depression and binge drinking. These treatments represent low-cost, wide-reach youth-appropriate treatment, which will have significantly public health implications for service design, delivery and health policy for this important age group.

**Trial registration:**

Australian and New Zealand Clinical Trials Registry ACTRN12614000310662. Date registered 24 March 2014.

## Background

Adolescence and young adulthood represents a critical period of development in which health and social behaviours are established. During this time, young people acquire a wide range of skills and behaviours via their family, social and community environments, and these influences will affect their physical, psychological and social health throughout their lives [1]. Early drinking during this period poses a risk later in life, with binge drinking during adolescence and young adulthood being associated with a 40–60 % increase in likelihood of developing alcohol dependence, regardless of family history [13]. Prevalence rates for alcohol use disorders in Australia are some of the highest worldwide [2]. Incidence rates for depression are much higher among young people than in any other age group [1], and at least 50 % of young people who experience a depressive episode will experience one or more recurrent episodes of depression [3].

Young people drink differently from adults. In Australia and the USA, 90 % of alcohol consumption in young people occurs in the form of binge drinking 5 or more standard drinks in one sitting, [4]. Epidemiological data indicate that binge drinking peaks at age 19, with the aim of getting drunk as quickly and cheaply as possible, in part to cope with a range of emotional issues present at that age [5]. Binge drinking in young people crosses all socioeconomic domains, and exerts its impact across all demographics and communities.

Mental disorders, particularly depression, will generally emerge during adolescence and young adulthood, and remain a significant challenge for our young people. Australian data indicate that 1 in 4 people aged 16–24 years will experience a mental health disorder in a 12 month period, and up to 43 % report feeling sad for at least 2 weeks in the past year [5]. Incidence rates for depression are much higher among young people than in any other age group, and at least 50 % of young people who experience a depressive episode will experience one or more recurrent episodes of depression [5].

Depression and binge drinking represent a lethal combination for young people, as evidenced by higher rates of land transport accidents and intentional self-harm than in any other age group 2 in every 5 deaths, [1]. Binge drinking is a common correlate of road accidents, and after a previous suicide attempt, depression is the next highest risk factor for youth suicide [1]. Heavy alcohol use, including binge drinking, plays a role in both the development and progression of mental disorders, particularly depression [6].

### Treatment access and retention of young people in traditional treatments is poor

There is hope of full functional, physical and psychological recovery from the consequences of depression and binge drinking. Animal models have shown that, with abstinence from a period of heavy binge drinking, neural stem-cell development can be re-instated, along with the formation of new neurons and other brain cells indicative of brain growth [7]. Effective treatment of depression during early days of abstinence from binge drinking can prevent the development of a depressive episode, and in young mice has been shown to better restore the ability of the brain to produce new cells [8].

In humans, psychological interventions, which allow the exploration of links between disorders, are of likely benefit to people with comorbid conditions, and are worthy of closer development and testing, in light of the potential for interactions between alcohol use and pharmacotherapy [9]. Psychological treatment is often preferred over pharmacotherapy [10], especially among young people [11]. Good interpersonal relationships and social connectedness have also been shown to exert a significant protective effect on the development of depression and in depressive and alcohol relapse [3, 12].

Despite this, treatment access for young people remains a critical challenge for traditional mental health and drug and alcohol services [13]. Patients under 25 years accounted for the least number of encounters with a general practitioner 2008–9 in Australia (20 %), representing a decrease in the frequency with which this age group is accessing primary care services since 1999–00 [1]. Only 2 % of 15–24 year olds accessed a psychologist in 2008–9, a rate that was half that of their older counterparts [1]. Similar patterns were seen for general counsellors.

### How can we encourage young people into treatment?

In 2015, 73 %, of Australia’s population were Internet users [14], with highest rates of access reported among young people 93 %, [15]. The internet has become an important source of health information for many people, with evidence indicating that 71 % of young people aged 18–29 search for health-related information online [16]. After e-mail and using search engines, searching for health information is the most popular online activity for adults [17].

The increased availability and use of internet-based programs as a supplement to health care is also a potential solution to well-documented treatment accessibility problems [18], particularly among people with depression and AOD use comorbidity. Treatment can be accessible at times and in locations that suit clients, without the need to schedule appointments or to confront the stigma associated with seeing a therapist. It offers privacy and anonymity, and allows clients to work at their own pace, tailoring the provision of information and strategies. Interactive and multimedia options offer the potential for higher levels of engagement than other self-help modalities [23]. A recent meta-analysis of internet-based interventions for alcohol misuse in adults revealed that they are highly effective in reducing alcohol consumption, and encouraging adherence to low-risk drinking guidelines [19]. Similarly in the depression field, internet-delivered cognitive behaviour therapy has demonstrated effectiveness in reducing depression, particularly when combined with support from a therapist [20]. We have previously demonstrated that, in an adult population, computerised treatment for comorbid depression and alcohol use problems was effective in reducing both depression and alcohol use simultaneously, and produced similar results to a face-to-face integrated intervention delivered by a psychologist [21]. A recent systematic review of treatments for co-occurring depression and substance use in young people highlighted the promise of psychological treatments targeting both issues simultaneously, and the need for more innovative techniques, such as Internet-based technologies, to better engage this group [22].

Social networking is also an increasingly popular online activity for young people, and is most common for Internet users in the younger age groups: 86 % of 18 to 24 year olds performed this activity in 2010–2011 [15]. Facebook is the most widely used of the social networking sites, and in Australia there are currently over 10,000 000 Facebook users, representing 51 % of the Australian population. The majority of these users are young people 12 % for 13–17 years; 24 % for 18 to 24 years; 26 % for 25–34 years, [23]. Results of a 2011 *Sensis®* survey of 803 randomly selected Australians reported that virtually all respondents aged under 30 (93 %) used social networking sites, and the majority accessed these sites every day [24]. Facebook is already frequently used by patients, carers and healthcare professionals to share their experiences with one another [25].

Given the popularity of social networking among young people, there is opportunity for identification and intervention regarding health behaviours particularly in the adolescent and young adult population [26]. Social support, integration, influence and social networks appear to play important roles in both behaviour change and emotional health [27]. Peers are considered more influential than parents in their contribution to adolescent binge drinking through such mechanisms as: modelling; interpersonal persuasion; shaping norms, attitudes, and values; and by providing opportunities and support for problem drinking [28, 29]. As a result, social networking websites have the potential to function as a platform for promoting and establishing (positive and negative) norms of behaviour among young people [30].

## Aims

The internet Treatment for Alcohol and Depression study (iTreAD, http://www.itread.com.au) will conduct the first randomised controlled trial of an Internet-delivered treatment for comorbid depression and binge drinking in young people aged 18–30 years, augmented with social networking support. We will examine the relative impact of:

- Online monthly self-assessments for 12 months (OSA);
- OSA + 12-months of access to a 4-week program of web-based intervention for binge drinking and depressed mood (DEpression ALcohol, DEAL);
- OSA + DEAL + 12-months access to a purpose-built social networking site (Breathing Space).

It is hypothesised that all groups will report reductions in binge drinking frequency and depression over the treatment period, but that this will be greatest for those receiving DEAL. It is also hypothesised that participants allocated to OSA + DEAL + Breathing Space will complete more web-based sessions and therefore report superior reductions in binge drinking frequency and depressive symptoms relative to the other treatment conditions.

### Primary hypothesis

(1) For binge drinking frequency and depression (DASS21) scores at 64 weeks post-baseline, OSA + DEAL + Breathing Space will have a superior impact over the other conditions.

### Secondary hypotheses

(2) Reductions in binge drinking frequency and DASS21 will occur at a faster rate in OSA + DEAL + Breathing Space than the other treatment conditions; and

(3) Participants in OSA + DEAL + Breathing Space will complete a greater number of DEAL sessions and self-assessments, and report greater retention than the other treatment conditions.

## Design

The iTreAD study is a randomised controlled superiority trial, with three parallel groups, and two primary endpoints (changes in depression and alcohol use between baseline and 64-weeks post-baseline assessment). The design is consistent with SPIRIT guidelines for clinical trials [31]. Ethics approval has been obtained from the University of New South Wales Human Research Ethics Committee (Approval #HC13299) as the primary site.

### Study setting

Participants will be sought across Australia, via online recruitment strategies (e.g. Facebook, Twitter, Google advertisements, other social media and online forums) and traditional methods (e.g. University campuses, general practitioner surgeries, health service settings, event location promotion). Screening, assessment, treatment and follow-up phases will be conducted in the community, via the Internet, at a time and location convenient to the participants.

### Inclusion and exclusion criteria

Inclusion criteria include: (1) aged 18–30 years; (2) current depressive symptoms score at least 5 on the DASS-Depression scale, [32]; (2) binge drinking at least 2 times in the previous month (≥5 standard drinks in the one sitting), as measured by the Alcohol Use Disorders Identification Test – consumption scale AUDIT-c, [33]; and (3) ability to access the Internet. Exclusion criteria are: (1) psychotic symptoms lasting >2 days; (2) non-English speakers; (3) people with organic brain diseases; and (4) serious risk of suicide.

### Participants

We will recruit 369 participants over a 24-month period (16/month). The trial has a purposive sample of young people reporting current binge drinking and comorbid depressive mood.

### Procedure

Initial contact with potential participants is by Internet, whereby they complete an initial screening assessment to determine eligibility and randomisation characteristics as per CONSORT guidelines [34]. Eligible participants receive an email link to online study information and consent processes; with access to the full online baseline assessment provided once consent is obtained. Randomisation to one of three treatment groups occurs upon online assessment completion. Following randomisation, participants are provided their login access code along with instructions about how to access the treatment website and log in. Independent, blind follow-up occurs across all conditions at 26, 39, and 52 and 64 weeks post-baseline.

### Screening

In response to project advertisements, potential participants complete an online screening tool that comprises the following instruments:

1. Entry criteria (age, Internet access);
2. AUDIT-c [33];
3. DASS21 [32];
4. Expectations of Online Treatment Questionnaire [35];
5. Independent/Interdependent Problem Solving Questionnaire [36];
6. Big 5 Inventory – Conscientiousness, Agreeableness, Neuroticism [37].

### Assessments

All assessment tools are widely administered in mental health and alcohol research, and were chosen to maximise compliance and minimise assessment burden (see Table 1).

**Table 1:** Assessments and administration frequency

Assessment Instruments	Baseline Assessment	Interim Online Self-Assessments	Independent Follow-up Assessment
		4–52 weeks post-baseline	26, 39, 52 & 64 weeks post-baseline
*Demographics*	✓	-	-
*Service utilisation*	✓	-	✓
*Depression*			
DASS21 [32]	✓	✓	✓
*Alcohol*			
AUDIT [33]	✓	-	✓
TLFB – Alcohol [45]	✓	✓ (past 2 weeks)	✓ (past month)
*Other*			
OTI – other drugs, energy drinks [46]	✓	-	✓
AQoL [47]	✓	-	✓
IPDEQ [48]	✓	-	-
Reactive-Proactive Aggression [49]	✓	-	✓
Psychiatric Symptom Frequency [50]	✓	-	✓
List of Threatening Experiences [51]	✓	-	✓
Impact of Event Scale – Revised [52]	✓	-	✓
CIDI – PTSD items [53]	✓	-	✓
	✓		-
*Client Satisfaction with Treatment*			
CSQ [54]	-	-	64 weeks

Demographics: information will include age, gender, area of residence (urban/rural), education and occupation, and ethnicity.

Service utilisation: includes self-reported hospitalisations, emergency room visits, attendance at clinics, rehabilitation programmes, contact with community mental health teams, psychologists, psychiatrists, other health professionals, use of general practitioners, and use of medication (including compliance). In addition, we will purchase Medicare data on consenting participants to objectively measure service utilisation throughout the treatment and follow-up period.

### Administration of follow-up assessments

The follow-up assessment team is located at a separate University to the baseline/treatment team to maximise blindness and independence. Follow-up assessors are blind to treatment allocation and facilitate completion of follow-up assessments via the Internet to all consenting participants regardless of treatment participation. Follow-up assessment occurs at 26, 39, 52 and 64 weeks post-baseline. There is good evidence that web-based assessments result in reliable and valid ratings of depressive and alcohol use disorders [38].

In line with best practice standards and our previous trial experience, the following strategies are employed to maximise retention in treatment and assessment: (1) obtain multiple contact modes at baseline; (2) rapid and assertive response if contact is temporarily lost (e.g. within 24 h); (3) solicitation of consent to continued assessment when participants wish to cease treatment; (4) negotiation of a reduced set of assessment core measures (i.e. of primary outcomes) when participants are unwilling to undertake the full follow-up protocol; (5) $20 AUD reimbursement for costs and time at baseline and a bonus ($20 AUD) for completing all scheduled follow-ups.

### Interventions

Access to the treatment website for each of the following conditions will be for a period of 12 months from the point of randomisation.

- Online Monthly Self-assessment (see Table 1, all 369 participants): Participants access the self-assessments from their preferred port for Internet access. Participants will receive an automated email prompt to log onto the website to complete their self-assessments at the required intervals. Information and referral to relevant services will be provided on the website to help the participant manage exacerbations in depressive symptoms and alcohol misuse. Participants will have a window of 2 weeks within which to complete each monthly self-assessment, before being designate as non-adherent to that month. Invitations for subsequent monthly assessments will occur regardless of previous compliance, through until the 12-month timepoint. There will be no further contact with the research group until the independent, blind follow-up commences.
- 4 sessions of web-based intervention (246 participants): Consists of 4 sessions of psychological treatment delivered entirely via the Internet (DEAL), using the preferred port for Internet access of the participants. Access to the DEAL (DEpression ALcohol) intervention is provided for a period of 12 months from randomisation. Content of the DEAL sessions include cognitive behaviour therapy (CBT) and motivational strategies [39]. Participants are asked to complete each session of the DEAL web-based intervention in sequence, a week apart, from the point of randomisation. The website tracks participants’ progress through each weekly module. In-built reminders prompt participants to access the DEAL program after periods of inactivity. Throughout the 12-month “treatment period” participants may review sessions of the DEAL program as desired.
- Breathing Space (123 participants): Access to a social networking community (Breathing Space) will be provided to participants allocated to this condition for a period of 12 months. The social networking platform is provided by CafeWell (Welltok Inc., https://www.cafewell.com), a web-based and mobile app that integrates support for behavioural and chronic health conditions, with access to moderated mental health support communities. Breathing Space will be created as a closed community on the CafeWell site, and moderated by Australian research clinicians associated with the project. During the hours of 9 am-9 pm, Australian research clinicians will encourage participants to post their thoughts, messages of support, update their status, tell their stories, share self-assessment results, and report on progress related to their mood and binge drinking (successes and challenges). Outside of these hours, USA-based coaches will monitor participants to ensure any crisis situation and/or inappropriate posts and situations are managed in a timely fashion. Notifications will be received via all participants’ nominated email addresses and mobile app when any member posts on the site, encouraging participants to view the post. Participants will create their own profile and alias, and will be encouraged not to use their real names. A strict code of conduct has been developed for people randomised to Breathing Space, which covers general conduct, privacy, confidentiality, discussion of past experiences, trauma and suicidal thoughts. The code of conduct attempts to strike a balance between allowing participants in the community to freely express the thoughts and feelings of relevance to them in a supportive and safe environment, whilst minimising the potential for social contagion, bullying, and other potentially damaging behaviours. In addition, a clinical moderator manual has also been developed, to guide the nature and content of interactions with participants in the Breathing Space community.

### Randomisation

Randomisation will be conducted independently of the study team, using an online program, which can only be accessed after eligibility is confirmed. Allocation will be on a 1:1:1 ratio, using permuted blocks of varying sizes (e.g. 3, 9) and stratified for gender and antidepressant status. Participants are advised of their treatment allocation once the study team is advised of their completed online baseline assessment.

### Primary endpoint

There are two primary endpoints: (1) depression at 64-weeks post-baseline, and (2) binge drinking frequency at 64-weeks post-baseline.

### Secondary endpoint

Secondary endpoints are at 64-weeks post-baseline, and include alcohol use, anxiety (DASS-anxiety), service utilisation, and quality of life (using the AQoL). Monthly self-assessment data will be used to track the rate of change in depressed mood and binge drinking frequency between baseline and 12-months post-baseline (the treatment period), to account for any change in symptoms as a function of the baseline assessment session.

## Statistical analysis plan

Data on screening, refusals and dropout are coded and reported as per CONSORT guidelines, and primary analyses use intention-to-treat. Preliminary analyses compare treatment groups for any baseline or health service utilisation differences using proportions for categorical variables and means/standard deviations for continuous variables.

The primary outcomes will be compared between groups at each time point using random effects linear regression that includes data from all time points. Separate models will be fitted for each outcome variable. The models will include the fixed effects of treatment (Online Monthly Self-assessment as referent category), time, the interaction between treatment and time as well as the baseline score and baseline score-by-time interaction. The primary contrast will be the difference in least squares means between treatment groups at week 64.

Since the standard errors for the fixed effects in the model depend on the variance-covariance structure that is used in the analysis, we will examine several possible structures including models with random slopes and intercepts, as well as a variety of error structures for the repeated measures (compound symmetry, AR1, Toeplitz). The model with the smallest Akaike Information Criterion (AIC) will be selected as our final model.

The mixed model provides unbiased estimates of the treatment effect under the assumption that data are missing at random (MAR). Since the possibility of non-ignorable missingness cannot be excluded, we will conduct sensitivity analyses using non-ignorable pattern-mixture and selection models to investigate the robustness of our conclusions across these different models for missing data.

Continuous secondary endpoints will be analysed using the model described above, dichotomous secondary endpoints will be modelled using logistic mixed models.

### Sample size and power calculations

Our previous trials indicate approximately 25 % attrition over a 52-week follow-up period [40]. Recruiting 123 participants per group (369 participants across 3 experimental groups) and assuming an attrition rate of 30 %, we anticipate having complete data from 87 participants per group at 64 weeks (*n* = 261).

Our projected sample size at 64 weeks provides sufficient statistical power (at the standard level of 0.80) to detect differences between treatment groups of 0.25 standard deviations or greater, with a type 1 error rate of 2.5 %. Using baseline data from our previous studies with the target population and the DEAL resource [41], this corresponds to a difference between conditions of 1.3 drinks/day, or 9.4/week, and of 2.4 on the DASS21. These differences are towards the lower limits of clinical significance. However, within-condition changes are likely to be substantial. Consistent with our previous research, differences between treatment groups of <0.25SD, provided there are equivalent retention rates and therapeutic alliance ratings, will not be considered clinically significant.

## Discussion

The iTreAD project focuses on a common clinical problem that causes substantial functional, economic, and health impacts; comorbid depression and binge drinking. These conditions are currently under-treated, contribute significantly to the global disease burden and are at their peak in young adulthood. Offering treatments of low cost and with wide reach to affected people will address current inequities of treatment access for these problems, and provide a youth-appropriate modality of treatment delivery. These results will have profound implications for service design and health policy, and speak to important questions about the nature of treatment effects in general.

In commencing the iTreAD study and engaging our study population, two key learnings have emerged, resulting in changes to the study design and delivery to better suit the needs of young people wanting to access treatment (via our study) for depression and binge drinking concerns.

1. Participant preferences for contact
  Our previous trials that have integrated Internet and computerised interventions into the clinical treatment of comorbid depression and addictive disorders, suggested that real time therapist-support was an influential factor in a potential participant’s decision to enrol in the study [21, 42]. Regardless of whether the treatment provided was entirely face-to-face with a therapist, or supplemented with a computerised treatment program, participants have previously reflected that more face-to-face support would have increased satisfaction with treatment [43]. Accordingly, the iTreAD methodology incorporated several points of “real-time” clinical contact with participants (via phone). For the 18–30 year age group, however, we have found the phone-based contact requirement to be a barrier to participation. For example, we had a significant proportion of participants who were eligible, and completed their online baseline assessment, but could not be contacted via telephone to speak with a clinician to complete their clinical interview, receive their randomisation, and commence the study treatments. Switching to a fully automated online consent and assessment process has seen significantly better retention and participation by the young people engaged in the trial to date, and progression through to randomisation and treatment for those young people who were unable to complete the phone-based ‘real time’ study phases.
2. Duty of care considerations in non face-to-face trials
  An important observation in the target population for the iTreAD study is that, despite setting the threshold for entry at relatively low levels of depression and binge drinking behaviours, study participants tend to be those reporting more severe depressive symptoms and regular (daily) alcohol use. By including additional optional mental health domains in our baseline assessment, we have also detected significant levels of exposure to traumatic events, and high levels of current trauma symptoms. This has presented an ethical and clinical challenge, given the nation-wide recruitment strategy, and the preference for non ‘real time’ contact by participants in our study. It has taken the combination of our expertise across Internet trials and significant clinical expertise in working with people with severe comorbidities to safely engage and support the young people in our trial. The online format for baseline assessment completion, whilst appealing to the age group, has also potentially provided the anonymity that might encourage higher levels of disclosure of traumatic events, severe symptoms, and high-risk behaviours than for traditional assessments. This is an important advantage of the Internet, however it also requires a considered and careful response to participants in managing these disclosures. We have thus devised a careful strategy to respond to these disclosures, which involves a range of ‘real time’ options (phone calls, personalised private messaging), and setting a threshold for self-report of suicidal and traumatic symptoms that automatically triggers a ‘real time’ response. Having clinically trained staff (e.g. clinical psychologists) is essential in these situations, as is regular clinical supervision sessions, and access to ‘real time’ expertise to work through options for follow-up, referral, and engagement of local crisis services to the participant should that be indicated.
3. Changes to the protocol since Clinical Trials Registration
  Eligibility Criterion for Depression: We have set eligibility for the current trial at a DASS-Depression scale of 5 or greater to ensure we recruit participants with current levels of depressive symptoms. Our clinical trials registration specifics an eligibility criterion for the DASS of 12 or greater on the full scale. This was modified as the criterion was not specific or sensitive enough to recruit our target sample.
  Removal of the Structured Clinical Interview for DSM SCID, [44]. With removal of the phone-based assessment, we also had to remove the SCID-Depression and SCID-alcohol scales from our baseline assessment, as these scales are not currently validated for delivery in self-report format. We will present SCID data on all participants who provided this information prior to the implementation of full online baseline assessment.
  Statistical Analysis Plan: since completion of the clinical trials registry, we have engaged the independent expertise of a designated statistician to develop the statistical analysis plan (and to carry out said plan) for the current trial. The plan reported in this study protocol is the analysis plan that will be undertaken at the conclusion of the study.
